# Human Antibody Production in Transgenic Animals

**DOI:** 10.1007/s00005-014-0322-x

**Published:** 2014-12-03

**Authors:** Marianne Brüggemann, Michael J. Osborn, Biao Ma, Jasvinder Hayre, Suzanne Avis, Brian Lundstrom, Roland Buelow

**Affiliations:** 1 Recombinant Antibody Technology Ltd., Babraham Research Campus, Babraham, Cambridge CB22 3AT UK; 2 Open Monoclonal Technology, Inc., Palo Alto, CA 94303 USA

**Keywords:** Transgenic animals, Antibody repertoires, Human epitopes, Class-switch recombination, B cell development, Locus silencing

## Abstract

Fully human antibodies from transgenic animals account for an increasing number of new therapeutics. After immunization, diverse human monoclonal antibodies of high affinity can be obtained from transgenic rodents, while large animals, such as transchromosomic cattle, have produced respectable amounts of specific human immunoglobulin (Ig) in serum. Several strategies to derive animals expressing human antibody repertoires have been successful. In rodents, gene loci on bacterial artificial chromosomes or yeast artificial chromosomes were integrated by oocyte microinjection or transfection of embryonic stem (ES) cells, while ruminants were derived from manipulated fibroblasts with integrated human chromosome fragments or human artificial chromosomes. In all strains, the endogenous Ig loci have been silenced by gene targeting, either in ES or fibroblast cells, or by zinc finger technology via DNA microinjection; this was essential for optimal production. However, comparisons showed that fully human antibodies were not as efficiently produced as wild-type Ig. This suboptimal performance, with respect to immune response and antibody yield, was attributed to imperfect interaction of the human constant region with endogenous signaling components such as the Igα/β in mouse, rat or cattle. Significant improvements were obtained when the human V-region genes were linked to the endogenous C_H_-region, either on large constructs or, separately, by site-specific integration, which could also silence the endogenous Ig locus by gene replacement or inversion. In animals with knocked-out endogenous Ig loci and integrated large IgH loci, containing many human Vs, all D and all J segments linked to endogenous C genes, highly diverse human antibody production similar to normal animals was obtained.

## Introduction

The first fully human monoclonal antibodies (mAbs) have been produced over 25 years ago by two parallel technologies: phage display, with selection of antigen-specific binders from blood lymphocyte libraries, and transgenic mice, with integrated human immunoglobulin (Ig) loci (Bruggemann et al. 1989a; Bruggemann and Neuberger 1996; McCafferty et al. 1990; Neuberger and Bruggemann 1997). In the transgenic approach, natural diversification and selection are being exploited as integrated loci are under the control of the animal’s immune system where they can undergo normal processes of DNA rearrangement and hypermutation. Interestingly, human or mouse, and probably all mammalian Ig loci work in very similar ways, which was confirmed in early experiments using a germline-configured chimeric construct (Bruggemann et al. 1989a).

A challenge to the transgenic antibody technology was the large size of the human Ig loci (Cook and Tomlinson 1995; Frippiat et al. 1995; Matsuda et al. 1993; Pallares et al. 1999; Weichhold et al. 1993). Emphasis was on inclusion of V genes, as many and as diverse as possible, rather than, for example, all D segments or several C genes for the H chain (reviewed in Bruggemann et al. 2007). Early approaches used minigenes and even with a small number of genes mAbs with good binding characteristics could be obtained (Green et al. 1994; Taylor et al. 1994; Wagner et al. 1994). Improvements in oocytes microinjection and the use of embryonic stem (ES) cells permitted the integration of larger regions; first on plasmids and cosmids (Bruggemann et al. 1991; Taylor et al. 1994), and then on bacterial artificial chromosomes (BACs) and yeast artificial chromosomes (YACs) (Davies et al. 1993; Mendez et al. 1997; Nicholson et al. 1999; Popov et al. 1999; Wagner et al. 1996).

At the same time, various Ig knock-out (KO) lines were derived, with µMT being the earliest mouse line with a silenced IgH locus (Kitamura et al. 1991). Apart from studying lymphocyte development and antibody expression in mice without fully functional H- and/or L-chain loci, these approaches aided human antibody expression from introduced transgenic Ig loci significantly. Many silencing strategies were successful: removal of the J_H_ segments, abolished DNA rearrangement (Chen et al. 1993a), deleting all C_H_ genes eliminated H chain expression and trans-switching (Ren et al. 2004), the various Cκ or Jκ KOs and a Cλ KO silenced IgL expression (Chen et al. 1993b; Sanchez et al. 1994; Zou et al. 2003). Breeding regimes to combine H- and L-chain KOs with human IgH and IgL transloci produced multi-feature animals expressing considerable levels of diverse fully human Ig.

**Fig. 1:**
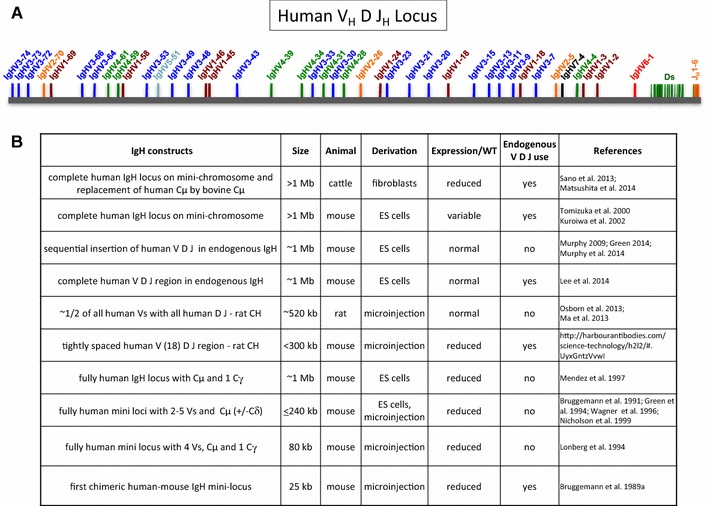
Human IgH loci. **a** The complete V_H_, D and J_H_ region from the most 5′ V_H_, IgHV3–74, to the most 3′ J_H_ segment, J_H_6, is accommodated on ~950 kb (Lefranc and Lefranc 2001). **b** Transgenic constructs and features. Animals were derived from manipulated fibroblasts (cattle) or ES cells (mouse) and by DNA microinjection (mouse and rat). In several lines, human Ig expression appeared to be reduced. Endogenous VDJ use or H chain products with non-human segments, such as mouse or cattle Vs, were obtained in strains with leaky or incomplete endogenous KO

The production of transgenic antibody repertoires, similar or comparable to those in humans, requires diverse rearrangement combined with high expression of human V, (D) and J segments, ideally from a large gene pool. This has been achieved only recently in rodent lines, not with fully human constructs but chimeric Ig loci, where immune responses similar to wild-type (WT) animals and diversity comparable to recorded human antibody sequences were found (Lee et al. 2014; Ma et al. 2013; Osborn et al. 2013). In Fig. 1a, the human IgH locus with all V_H_s, Ds and J_H_ genes/segments is illustrated. Features of the various fully human and chimeric IgH transloci, large and small, are presented in Fig. 1b.

## Fully Human Ig Transloci

Fully human transloci contain exclusively human V, (D), J and C genes, while in chimeric human transloci human V, (D) and J genes or regions have been linked to host (rat, mouse or cattle) C genes. In many cases, the size of the fully human Ig constructs assembled, for example, in plasmids, cosmids or phages, limited the number of genes that could be included as these vectors could only accommodate regions below ~100 kb. This meant that V genes had to be gathered in close proximity, D and J segments were tightly linked and C genes had to be carefully chosen. The result was poor expression from such mini-gene constructs although several useful human antibodies have been derived (Lonberg et al. 1994). The assembly of larger constructs in YACs by homologous overlaps (Davies and Bruggemann 1993; Davies et al. 1992; Popov et al. 1996), and subsequent introduction of YACs into ES cells by spheroplast fusion or lipofection enabled the integration of several hundred kb up to ~1 Mb large human IgH and IgL regions into the mouse genome (Choi et al. 1993; Davies et al. 1993; Mendez et al. 1997; Nicholson et al. 1999; Popov et al. 1999). Introduction of human mini-chromosomes by microcell-mediated chromosome transfer established ES cell-derived mouse lines producing fully human mAbs (Kuroiwa et al. 2002; Tomizuka et al. 2000) and cattle, derived from manipulated fibroblasts, which expressed considerable amounts of human polyclonal Ig (Matsushita et al. 2014; Sano et al. 2013). However, the production of fully human mAbs in transchromosomic mice appeared to be less than 1 % (Tomizuka et al. 2000) and only about 50 % of fully human IgG was identified in transchromosomic cattle, often at quite reduced levels (Matsushita et al. 2014). Although problems concerned with trans-chromosome truncation, chimerism and transmission stability have been addressed, these could not be fully resolved (Kakeda et al. 2005). A solution, which considerably improved expression and efficiency of hybridoma production, was cross-breeding of IgH trans-chromo mice with Igκ XenoMouse (Ishida et al. 2002; Mendez et al. 1997).

Despite the improvement and breeding with endogenous KO lines, human antibody titers were still suboptimal or at best variable and despite diverse V, D and J usage, class-switching was frequently poor and hypermutation was relatively low (Bruggemann et al. 2007). A problem for many academic researchers was that supposedly superior transgenic animals, produced by companies, were not generally available and little support was provided to improve available lines or constructs once they had been published or shown to work in principle. Nevertheless, useful fully human IgM antibodies of reasonable affinity against HIV (when converted to human IgG1) could be retrieved even from lines carrying few V genes and lacking class-switching (Pruzina et al. 2011).

Rearrangement and expression of human IgL loci was achieved with mini-gene constructs assembled on plasmids and also larger regions on BACs and YACs; these earlier constructs have been extensively reviewed (Bruggemann et al. 2007, 2014). A comparison of human Igκ L-chain transloci showed that various germline-configured regions, from 15 kb to over 1 Mb, readily express serum Ig but that larger regions, with naturally spaced genes, produce much higher antibody yields (Xian et al. 1998). Several larger fully human IgL loci were highly expressed even in a WT background, which showed successful competition with the endogenous L-chain loci. For example, an authentic 380 kb human Igλ YAC with 15 functional Vλ genes produced high antibody titers from diverse and hypermutated transcripts (Nicholson et al. 1999; Osborn et al. 2013; Popov et al. 1999). Thus, it can be concluded that large fully human L-chain constructs can be expressed similar to WT L-chain levels in transgenic animals (Ma et al. 2013; Osborn et al. 2013).

## Chimeric Human Ig Transloci

To ensure optimal physiological interaction of translocus-encoded membrane Ig with endogenous components of antigen-receptor associated polypeptides, it has been speculated and later shown that chimeric constructs with human VDJ linked to rodent C_H_ are most efficient (Green 2014; Ma et al. 2013; Osborn et al. 2013; Pruzina et al. 2011). Strategies to link human IgH V genes, D and J segments with mouse or rat C_H_ genes made use of BAC and YAC technology for (1) sub-cloning and joining large regions to secure overlapping integration by DNA microinjection into oocytes (Ma et al. 2013; Osborn et al. 2013) and (2) sequential site-specific integration of BACs with human V and V-D-J regions upstream of mouse Cµ by gene targeting or recombinase-mediated exchange (Lee et al. 2014; Murphy 2009). Human V_H_s are efficiently rearranged in transgenic mammals and their linkage to endogenous C_H_ genes provides a flawless interplay with the cellular signaling machinery. Combined interactions of the many components that aid B cell receptor (BCR) signaling (see: http://www.biocarta.com/genes/PathwayGeneSearch.asp) secure efficient B cell activation with normal immune responses. Endogenous C_H_ genes will also allow optimal physiological interactions necessary in the regulation of immune responses by the various host Fc receptors (Nimmerjahn and Ravetch 2007; Rowland et al. 2013).

Conventional transgenic technologies with DNA microinjection into oocytes enable the integration of multiple BACs and large YACs. This allowed the insertion of three linear and overlapping BAC regions with homologous end sequences 5′ and 3′, which provided about half of all human V genes, all D and all J segments (Osborn et al. 2013). A comparison of independent founders carrying a continuous region of over 500 kb randomly integrated found high expression levels independent of the site of integration. The conclusion derived from many chimeric (human V D J, rat C) IgH, as well as fully human Igκ and Igλ lines, is that antibodies obtained from multiple large integrations at one position can be as efficiently expressed as WT Ig if the constructs contain the necessary regulatory sequences (Ma et al. 2013). Furthermore, multiple integration of microinjected DNA, i.e., several copies of the same BAC or YAC, may well be advantageous as larger gene pools with an increased number of available genes could facilitate more extensive receptor editing (Liu et al. 2013; Nemazee and Weigert 2000; Ouled-Haddou et al. 2014; Zhang et al. 2003).

Considerable efforts have been made to integrate large numbers and possibly all human V_H_, D and J_H_ segments upstream of mouse Cµ (Green 2014; Lee et al. 2014; Murphy 2009; Murphy et al. 2014). Lee et al. (2014) showed that with site-specifically integrated WT and mutated *loxP* sites, it was possible to provide a BAC landing pad for adding human V-regions upstream of the first endogenous C gene. This approach employed sequential integration of modified BACs, which reconstituted or replaced the mouse V_H_-region with equivalent human genes. The result was to reconstitute a near authentic human V_H_-D-J_H_ as well as Vκ-Jκ and Vλ-Jλ region (Green 2014; Lee et al. 2014; Murphy 2009). With these insertions, duplications or multimeric integration of the same BAC are barred, which ensures a genuine V gene order and content. However, there has been no indication that non-selected or random integration of large transloci was disadvantageous and choosing particular founders (or combination of founders for IgH and IgL) secured high expression and breeding to homozygosity.

Integration of human L-chain V-J regions adjacent to the mouse C_L_ gene has also been shown to yield extensive chimeric antibody repertoires, but reports explained that endogenous L-chain rearrangements were not entirely prohibited (Green 2014; Lee et al. 2014). In contrast, randomly integrated fully human Igκ or Igλ transloci could be cleanly expressed—without residual rodent Ig in KO lines—and extensive levels and diversity similar to WT have been obtained (Osborn et al. 2013). This means that there are no apparent advantages employing locus replacement strategies in this instance.

## Ig KO Strains

Ig loci have been knocked out or disabled in mouse, rat and cattle (Bruggemann et al. 2007; Matsushita et al. 2014; Osborn et al. 2013; Tomizuka et al. 2000). Strategies involved gene targeting in ES cells via insertion or deletion using, for example, Cre/loxP which allowed the removal of >100 kb regions (Ren et al. 2004; Zou et al. 2003). Recently, the use of zinc-finger (endo)nuclease (ZFN) constructs for DNA microinjections into oocytes produced several IgH and IgL KO lines in the rat (Geurts et al. 2009; Menoret et al. 2010; Osborn et al. 2013) and silenced the IgH locus in rabbits (Flisikowska et al. 2011). Advantages of the ZFN technology are that non-homologous end joining to silence a gene or locus via deletions up to several kb can also provide a target site for homologous integration (Cui et al. 2011).

More recently combined gene targeting and locus extension have been taken to the next level. In one approach where Cre recombinase was directed to opposite *loxP* sites, integration was followed by inversion of the mouse V_H_-D-J_H_ region and, separately, Vκ-Jκ region (Lee et al. 2014). This would not necessarily prevent DNA rearrangement but with sufficient separation from C genes splicing resulting in V(D)J-C products appeared to be minimal (Lee et al. 2014). In another approach, deletion of the whole V-region from both mouse IgH and Igκ loci was obtained (Green 2014).

A considerable advantage in expressing a human antibody repertoire in transgenic animals is the lack of all endogenous Ig as this greatly simplifies the production of polyclonal Ig, as shown in cattle (Matsushita et al. 2014), and monoclonal Ig in rats (Osborn et al. 2013) and mice (see: http://www.crescendobiologics.com/rnd/crescendo-mouse). In these animals, no IgH, Igκ and Igλ with endogenous V(D)J is being produced. The absence of endogenous Ig prevents a bias in the immune response, for example, selecting more compatible mouse idiotypes, and means that selection and purification can be avoided.

## Applications

mAb therapy has been developed to provide reagents that specifically bind to cellular receptors or interact with circulating targets for their removal or neutralization. Target cell depletion can be achieved by Fc receptor interaction and antibody-dependent cellular cytotoxicity has been identified as an important mechanism to eliminate tumor cells (Hjelm et al. 2006; Kim and Ashkenazi 2013; Mellor et al. 2013). Binding of mAbs to particular cell-surface receptors via the variable domain and simultaneously engaging with natural killer cells, monocytes or macrophages via Fc receptor interaction provided by the constant region (C region) initiates a cascade of events important for removal or reduction of abnormal or malignant cells and pathogens. In many epithelial tumors, epidermal growth factor receptor is expressed at high levels and the tumor cell load of many patients could be reduced with Vectibix (see: http://www.vectibix.com). Tumor inhibition was also achieved with anti-CD20 mAbs, which were effective in initiating the destruction or killing of B cells from patients with chronic lymphocytic leukemia, another very aggressive disease (Zhang 2009).

Important validation for using transgenic lines includes how well they compare with WT animals with respect to serum titer, diversity and B cell development and if they can be bred to homozygosity, which was achieved with various rat and mouse strains. Success in DNA rearrangement and antibody expression of the first construct assembled in germline configuration (Bruggemann et al. 1989a) was followed by important progress regarding gene targeting, to knock-out the mouse Ig loci, and genetic engineering, to assemble large regions on artificial chromosomes such as YACs (reviewed in Bruggemann 2001). These advances were crucial to obtain specific, fully human antibodies from the introduced human IgH (mini)loci expressed in mice in the 1990s (Fishwild et al. 1996; Lonberg et al. 1994; Mendez et al. 1997). However, none of these early strains could match WT animals with regard to B cell numbers, antibody titers, diversity and hypermutation (Bruggemann et al. 2014; Green and Jakobovits 1998). Nevertheless, despite reduced expression levels, antibodies from these early transgenic animals, in clinical trials and on the market, have been obtained using hybridoma technology (Green 2014). To overcome the limitations of these first-generation strains, new transgenic animals have been designed (Lee et al. 2014; Ma et al. 2013; Osborn et al. 2013).

FDA approved and marketed fully human mAbs produced from the early mouse strains are listed in Table 1. The success of these mouse lines accelerated the shift from using murine and chimeric antibodies to fully human antibodies (reviewed in Bruggemann et al. 2014). Initially, conventional immunization regimes and myeloma fusion with spleen cells were adopted from procedures using WT mice or rats (Kohler and Milstein 1975; Lachmann et al. 1980). However, in recently derived strains, mAb production was significantly increased by the optimization of immunization protocols. For example, fusions of mouse myeloma cells with rat iliac lymph node cells collected after rapid immunization schemes and, separately, spleen cells obtained after multiple or booster immunizations, frequently resulted in many thousand hybridomas from one animal with sometimes over 100 diverse and high-affinity IgGs (Osborn et al. 2013).

**Table 1:** FDA approved fully human therapeutic monoclonal antibodies

Year	Name	Target	Treatment	Transgenic line (references)
2011	Yervoy (ipilimumab)	CTLA-4/CD152	Melanoma	Lonberg et al. (1994)
2010	Prolia (denosumab)	RANKL	Osteoporosis	Mendez et al. (1997)
2009	Simponi (golimumab)	TNF-α	Rheumatoid arthritis	Lonberg et al. (1994)
2009	Arzerra (ofatumumab)	CD20	Lymphocytic leukemia	Fishwild et al. (1996)
2009	Stelara (ustekinumab)	IL-12/IL-23	Psoriasis	Lonberg et al. (1994)
2009	Ilaris (canakinumab)	IL-1β	Auto-inflammation	Lonberg et al. (1994)
2006	Vectibix (panitumumab)	EGFR	Colorectal cancer	Mendez et al. (1997)

In 2014, a large number of defined (see: http://en.wikipedia.org/wiki/List_of_monoclonal_antibodies) and non-disclosed (see: http://biz.yahoo.com/e/130503/regn10-q.html) fully human antibodies were being evaluated in clinical trials. Close to 500 mAbs have been reported to be in active clinical development during the past 3 years, including ~25 % in phase I, ~40 % in phase II, ~20 % in phase III and ~15 % at approval stage (BioPharm Insight database). A large majority, ~60 %, are indicated primarily for treatment of cancer, e.g., reduction of solid tumors possibly by blocking growth or inducing apoptosis. Other therapeutic categories include immunological (10 %), musculoskeletal (7 %) and infectious diseases (4 %). Therapeutic antibodies are also being developed for respiratory, dermatologic, central nervous system, hematologic, eye/ear, cardiovascular, HIV, liver and other diseases. Clinical-stage antibodies derived from transgenic animals are shown in Table 2. Four distinct mouse lines have been used to generate these: HuMAb antibody or UltiMAb antibody Mouse (Lonberg et al. 1994), XenoMouse (Mendez et al. 1997), TransChromo Mouse (Ishida et al. 2002) and VelocImmune Mouse (Murphy et al. 2014).

**Table 2:** Promising fully human antibodies in clinical trials in 2014

Name	Target	Treatment	Transgenic line (references)
Actoxumab/bezlotoxumab	Clostridium difficile	Clostridium difficile infection	Lonberg et al. (1994)
Alirocumab	Neural apoptosis-regulated proteinase 1	Hypercholesterolemia	Murphy et al. (2014)
Anifrolumab	IFN-α/β receptor	Systemic lupus erythematosus	Lonberg et al. (1994)
Brodalumab	IL-17	Inflammatory diseases	Mendez et al. (1997)
Conatumumab	TNF-related apoptosis-inducing ligand	Solid tumors Cancers of hematopoietic origin	Mendez et al. (1997)
Daratumumab	CD38	Multiple myeloma	Lonberg et al. (1994)
Dupilumab	IL-4Rα	Allergic disease	Murphy et al. (2014)
Eldelumab	IFN-γ-induced protein	Crohn’s disease ulcerative colitis	Lonberg et al. (1994)
Enoticumab	DLL4	Solid tumors	Murphy et al. (2014)
Evolocumab	PCSK9	Hyperlipidemia	Mendez et al. (1997)
Fulranumab	NGF	Pain	Mendez et al. (1997)
Fasinumab	Nerve growth factor	Pain	Murphy et al. (2014)
Ganitumab	IGF-1	Cancer	Mendez et al. (1997)
Guselkumab	IL-13	Psoriasis	Mendez et al. (1997)
Inclacumab	Selectin P	Cardiovascular disease	Lonberg et al. (1994)
Intetumumab	CD51	Solid tumors	Murphy et al. (2014)
Iratumumab	CD30	Hodgkin’s disease	Lonberg et al. (1994)
Lirilumab	KIR2D	Solid tumors	Lonberg et al. (1994)
Lucatumumab	CD40	Lymphoma	Mendez et al. (1997)
Necitumumab	EGFR	Non-small cell lung carcinoma	Lonberg et al. (1994)
Nesvacumab	Angiopoietin 2	Cancer	Murphy et al. (2014)
Nivolumab	PD-1	Cancer	Lonberg et al. (1994)
Patritumab	HER3	Non-small cell lung cancer	Tomizuka et al. (2000)
REGN1033	Myostatin (GDF8)	Metabolic disorders	Murphy et al. (2014)
Rilotumumab	HGF	Solid tumors	Mendez et al. (1997)
Sarilumab	IL-6	Rheumatoid arthritis	Murphy et al. (2014)
Secukinumab	IL-17A	Uveitis Rheumatoid arthritis Psoriasis	Mendez et al. (1997)
Teprotumumab	IGF1R/CD221	Hematologic tumors	Lonberg et al. (1994)
Ticilimumab/tremelimumab	CTLA-4	Cancer	Mendez et al. (1997)
Urelumab	4-1BB/CD137	Anti-tumor	Lonberg et al. (1994)
Vantictumab	Frizzled 7 receptor	Solid tumors Breast cancer	Murphy et al. (2014)
Zalutumumab	EGFR	Solid tumors	Lonberg et al. (1994)
Zanolimumab	CD4	T cell lymphoma	Lonberg et al. (1994)

Different transgenic species are vitally important in providing human antibodies because of the trans-species gene similarities. Thus, sequence comparison of rat, mouse and human demonstrates that some genes are strikingly similar and that the less similar animal provides the better immune response. The other advantage of using different transgenic species (e.g. rats and mice) is that their transgene usage varies. As a result, many more genes from different families yield antibodies of high affinity for the same antigen, which may prove valuable for future immune therapy with mAb mixtures. For example, high-affinity human antibodies have recently been obtained from several other rodent lines; OmniRat (Ma et al. 2013; Osborn et al. 2013) and OmniMouse (http://www.omtinc.net), Kymouse (Lee et al. 2014) and Harbour Mouse (http://harbourantibodies.com/science-technology/h2l2/#.UyxGntzVvwI). Rabbits have also been used to produce human antibodies (Flisikowska et al. 2011). Novel antibodies with a common light chain have been obtained from the MeMo mouse (http://www.merus.nl/technology/memo.html) and OmniFlic (http://www.openmonoclonaltechnology.com/antibody_technology.html) lines. For therapeutic applications and minimizing immunogenicity, endogenous C regions can be easily replaced by the desired isotype without compromising antigen specificity (Bruggemann et al. 1989b; Presta 2006).

## Conclusions

Human antibodies produced in transgenic animals represent an important, broad and rapidly growing therapeutic class with a predicted global market reaching many billion US dollars (http://www.marketresearch.com). The success is based on a now proven technology with DNA insertion of Ig loci into the germline followed by expression of human antibody repertoires. Transgenic animals are increasingly used to produce antibodies with fully human idiotypes, a trend that is expected to continue in years to come as described in reports by The Times and BBC news (both June 3, 2014) as “trials show they are fulfilling their promise fast”.

Significant improvements have been made recently with the derivation of transgenic rat and mouse lines that, after immunization, readily produce diverse high-affinity antibodies as efficiently as WT animals (Osborn et al. 2013). World-leading biopharmaceutical companies are now using these animals to generate future human therapeutic antibodies for a wide range of conditions.

## Abbreviations

BAC: Bacterial artificial chromosome
C-region: Constant-region
D: Diversity segment
EGFR: Epidermal growth factor receptor
H: Heavy chain
Ig: Immunoglobulin
J: Joining segment
L: Light chain
mAb: Monoclonal antibody
V: Variable gene
WT: Wild type
YAC: Yeast artificial chromosome
ZFN: Zinc-finger nuclease
